# Recombinant MS087-based indirect ELISA for the diagnosis of *Mycoplasma synoviae*

**DOI:** 10.3389/fvets.2024.1472979

**Published:** 2024-10-29

**Authors:** Yang Zhang, Yan Wu, Jiawei He, Jiacui Lai, Honglei Ding

**Affiliations:** Laboratory of Veterinary Mycoplasmology, College of Veterinary Medicine, Southwest University, Chongqing, China

**Keywords:** *Mycoplasma synoviae*, subcellular localization, working condition, reproducibility, cross-reactivity, sensitivity, specificity

## Abstract

Accurate detection is a prerequisite for effective prevention and control of *Mycoplasma synoviae* infection. ELISA is the most popular method for the clinical detection of *M. synoviae* because of its convenience, low cost, and high detection rate. However, the cross-reactivity of commercially available ELISA kits with other avian pathogen-positive sera needs to be addressed. The aim of this study was to establish an ELISA method with high specificity for the detection of anti-*M. synoviae* antibodies in chicken serum to evaluate the *M. synoviae* infection status on poultry farms. The recombinant MS087 (rMS087) protein was expressed in *Escherichia coli* BL21 (DE3) and purified by Ni^2+^ affinity chromatography. An antibody against rMS087 was generated by immunizing BALB/c mice. Bioinformatic analysis revealed that MS087 was conserved among *M. synoviae* strains. Western blotting and indirect immunofluorescence results indicated that MS087 was not only localized in the cytoplasm and on the membrane but also secreted by the organism. For the established ELISA method based on rMS087, the optimal antigen concentration, blocking buffer, blocking duration, serum dilution, serum incubation duration, secondary antibody dilution, secondary antibody incubation duration and colorimetric reaction duration were 2 μg/mL, 1% BSA, 3 h, 1:500, 1.5 h, 1:20,000, 2 h and 5 min, respectively. Validation of the rMS087-based ELISA revealed a cut-off value of 0.5. The coefficients of variation of both the intra-batch and inter-batch methods were less than 9%. The assay was able to differentiate positive serum against *M. synoviae* from antisera against nine other avian pathogens and was able to recognize *M. synoviae*-positive sera at a dilution of 1:1,000. Compared with the commercial ELISA method, the rMS087-based ELISA has the potential to recognize more positive sera against *M. synoviae*. Collectively, the rMS087-based ELISA is a reproducible, specific, and sensitive serological method for detecting antibodies against *M. synoviae* in chicken serum and has robust potential for large-scale serological epidemiology of *M. synoviae* infection on poultry farms.

## 1 Introduction

*Mycoplasma synoviae* is a widespread pathogen in the poultry industry. It was first reported to be associated with the occurrence of infectious synovitis in chickens in the USA in the early 1950s (1) and was proven to be the causative organism for hemagglutination of red blood cells (2). In addition to acute/chronic respiratory disease, air sacculitis and/or articular lesions (3, 4), *M. synoviae* infection often results in reduced growth, production, and hatchability (5). Moreover, many studies (6–8) have described the association between the presence of *M. synoviae* in the oviduct and the production of eggs with eggshell apex abnormalities (EAA) by laying hens, characterized by an altered shell surface, shell thinning, increased translucency (detectable macroscopically, particularly upon candling), and the occurrence of cracks and breaks. *M. synoviae* is transmitted both horizontally and vertically, and its prevalence appears to be increasing worldwide (9). Since 2010, this pathogen has been widely prevalent in broiler flocks in mainland China (10, 11) and has subsequently rapidly spread to layer flocks (12).

Generally, *M. synoviae* infection can be controlled by three general approaches: biosecurity measures, medication with antimicrobials, and vaccination with commercial or autogenous vaccines (9). Several studies reported a temporary effect of antimicrobial treatments in EAA-affected layer flocks, with a decreased number of broken or downgraded eggs during treatment, but a disappearance of this effect 1–2 weeks after the end of treatment (6, 13) because the organism entered cells after infection (14–16). Although the live vaccine (MS-H) developed in Australia alleviates clinical symptoms and pathological damage and improves production performance in chickens (17, 18), it is used only in *M. synoviae*-free flocks and cannot block infection by wild-type strains (19, 20). Therefore, eradication measures, combined with biosafety regulations, constitute most cost-effective strategy for preventing and controlling *M. synoviae* infection.

In general, the most crucial step for the eradication of infectious disease is the use of appropriate diagnostic reagents. Serological tests are considered indispensable and cost-effective tools. Several serological tests, including rapid plate agglutination (RPA), hemagglutination inhibition (HI), and enzyme-linked immunosorbent assay (ELISA), have been developed for monitoring *M. synoviae* infection in chicken flocks (21–23). ELISA has been reported to have higher specificity than RPA and higher sensitivity than HI (21). Several ELISAs based on whole cells or membrane proteins have been developed to detect antibodies against *M. synoviae* (21, 24–26). However, the cross-reactivity and nonspecific reactions of these ELISAs with *Mycoplasma gallisepticum* have impeded the development of specific serodiagnostic tests (21, 25, 26).

A more specific ELISA was developed by using the MSPB protein, which is cleaved from the amino terminus of VlhA (27, 28), and the cross-reactivity of the method with sera against *M. gallisepticum* was overcome (28). However, the coating antigen shows a high degree of amino acid variability between strains (29) or even clonal isolates from a single strain (30), which affects the sensitivity of the established ELISA (27, 28). Recently, the membrane protein LP78, which binds to fibronectin and plasminogen, was used as the diagnostic antigen. Compared with commercial ELISA kits, although no cross-reactivity was observed with other poultry pathogen-positive sera, especially *M. gallisepticum*-positive sera, LP78-based ELISA demonstrated lower sensitivity in the detection of *M. synoviae*-positive serum samples (31). Therefore, it is necessary to develop a novel serological method with good specificity and sensitivity for the diagnosis of *M. synoviae* infection.

In general, membrane proteins are commonly used as targets for serological diagnoses. We found a *M. synoviae* protein MS087, which is predicted to be an F1-like ATPase-associated subunit (32), was localized in both the cytoplasm and membrane and is even secreted from the organism. In this study, we used MS087 as the coating antigen to develop an indirect ELISA to detect antibodies in chicken serum against *M. synoviae*, and provide a specific tool for the investigation of the epidemiology of *M. synoviae* in chicken farms.

## 2 Materials and methods

### 2.1 Bacterial strains, plasmid, sera, and culture conditions

*M. synoviae* strain CQTL01 was isolated from the synovial fluid of a Three-Yellow broiler in China in 2022. The strain was subsequently grown in KM2 medium (Tuopu, Zhaoyuan, Shandong, China) supplemented with 20% porcine serum (Jianglai, Shanghai, China) and 0.01% NAD (Sangon Biotech, Shanghai, China) at 37°C. The *Escherichia coli* strains DH5α and BL21(DE3) were grown in Luria–Bertani (LB) broth or on solid medium. The pET-30a(+) expression vector was preserved by our laboratory. Three hundred and sixty-eight serum samples were collected from five commercial poultry farms and two poultry slaughterhouses and were assessed with a *M. synoviae* ELISA antibody test kit (IDEXX, Westbrook, ME, USA) (Table 1). Chicken sera against other avian pathogens, including *M. gallisepticum*, *Avibacterium paragallinarum* (AP) serovars A, B and C, *Salmonella* Pullorum-Gallinarum (SPG), Newcastle disease virus (NDV), and avian influenza virus (AIV) subtypes H5, H7 and H9, respectively, were purchased from Harbin National Engineering Research Center of the Veterinary Biologics Corp in China.

**Table 1:** Information on chicken sera collected from five poultry farms and two poultry slaughterhouses.

Poultry farm/slaughterhouse	Sera from broilers		Sera from laying hens		Total
	Positive	Negative	Positive	Negative	
Poultry farm 1	0	0	0	23	23
Poultry farm 2	64	0	0	0	64
Poultry farm 3	11	0	0	0	11
Poultry farm 4	23	1	0	0	24
Poultry farm 5	20	0	0	0	20
Slaughterhouse 1	37	2	3	58	100
Slaughterhouse 2	0	0	33	93	126
Total	155	3	36	174	368

### 2.2 Bioinformatic analysis

The full-length sequence of the gene encoding *ms087* in the CQTL01 strain was obtained from the genome sequence (The data have been deposited in China National Center for Bioinformation, run accession: CRR1309196). The molecular weight (MW) of MS087 was computed with Detaibio.^1^ BLASTP^2^ was used to carry out amino acid identity matching with sequences retrieved from the NCBI database. The predictor SignalP 6.0 (33) was used to detect the presence of the signal peptide. TMHMM 2.0 (34) was applied to predict transmembrane helices. The computational online software programs CELLO v.2.5 (35) and Gpos-mPLoc (36) were used to directly predict the subcellular localization of the protein. The immunogenicity of the protein was calculated by VaxiJen V2.0 (37) using the default parameters.

### 2.3 Cloning, expression and purification of *M. synoviae* rMS087

On the basis of the *ms087* gene sequence from the CQTL01 strain, two primers (ms087-F: CGCGGATCCATGAAAATAAAAAAACT TTTATCTTTTGC and ms087-R: CCGCTCGAGATCATTTGC AAAATTAGTTAAATAAGT) were designed and synthesized. The genomic DNA of the CQTL01 strain was extracted using a TIANamp bacteria DNA kit (Tiangen, Beijing, China). The *ms087* gene was amplified with *ApexHF* HS DNA Polymerase FS Master Mix (Accurate, Changsha, Hunan, China) by using the genomic DNA of CQTL01 as the template. After purification, the PCR product was digested with *Bam*H I and *Xho*I and ligated to the expression vector pET-30a(+). The recombinant plasmid was subsequently transformed into *E. coli* DH5α and BL21 (DE3) competent cells via the heat shock method. Recombinant MS087 (rMS087) was expressed by induction with 1 mmol/L isopropyl *β*-D-1-thiogalactopyranoside (IPTG) at 16°C for 20 h on a shaker at 200 r·min^−1^. After induction, the expression and expression form of rMS087 in the recombinant bacteria were examined via 12% sodium dodecyl sulfate–polyacrylamide gel electrophoresis (SDS–PAGE) and Western blotting with a mouse anti-His tag antibody (Bioss, Beijing, China). Soluble rMS087 was purified with Ni-NTA HisTrap™HP (Cytiva, Shanghai, China), and the concentration of purified recombinant protein was measured via a BCA protein assay kit (Epizyme Biotech, Shanghai, China) according to the manufacturer’s instructions.

### 2.4 Raising polyclonal antisera against rMS087

Fifty micrograms of purified rMS087 protein at a concentration of 1 mg/mL emulsified with an equal volume of Freund’s complete adjuvant (BioFROXX, Beijing, China) was used to immunize female BALB/c mice aged 7 weeks via multipoint subcutaneous injection into groins and back. Booster dose of 50 μg of emulsified rMS087 protein with an equal volume of Freund’s incomplete adjuvant were applied on days 21 and 28 after the first immunization, and additional booster dose of 50 μg of purified rMS087 protein was applied on days 35 and 42 after the first immunization, respectively. The day before each immunization, blood samples were collected from the retro-orbital sinus. On day 49 after the first immunization, blood samples were collected from the eyeballs and the mice were euthanized. A specific antibody against rMS087 in sera were evaluated via indirect ELISA. Briefly, 96-well ELISA plates (Corning Incorporated, Kennebunk, ME, USA) were coated with 100 μL of purified rMS087 protein (0.5 μg/mL) in 0.05 mol/L carbonate buffer (pH 9.6) overnight at 4°C after incubation at 37°C for 1 h. Then, the plates were blocked with 5% skim milk diluted with PBS and subsequently incubated with serially diluted sera (from 1:500 to 1:2,048,000). The produced antisera were used for identification of the distribution of MS087 in *M. synoviae*. The protocols were approved by the Institutional Animal Care and Use Committee of Southwest University (IACUC No. IACUC-20240322-01).

### 2.5 Identification of the subcellular localization of MS087 in *M. synoviae*

To determine the distribution of MS087 in *M. synoviae*, an indirect immunofluorescence assay (IFA) and Western blotting were performed. Suspension IFA was performed as previously described (38). Briefly, 50 mL of *M. synoviae* strain CQTL01 cultured in the late logarithmic growth phase was collected and washed three times with PBS by centrifugation. Afterward, the cells were incubated with mouse anti-rMS087 polyclonal antiserum or preimmune serum diluted at 1:10,000 with PBS containing 0.5% bovine serum albumin (BSA) overnight at 4°C with shaking at 70 r·min^−1^. After washing, the cells were incubated with a 1:300 dilution of CoraLite488-conjugated goat anti-mouse IgG(H + L) (Proteintech, Wuhan, Hubei, China).

The cytoplasmic and membrane fractions of *M. synoviae* strain CQTL01 were separated using a membrane and cytoplasmic protein extraction kit (Biosharp, Hefei, Anhui, China) according to the manufacturer’s protocol. The supernatant of the culture medium after centrifugation was also collected. The cytoplasmic fraction and the culture supernatant were concentrated with a membrane and cytoplasmic protein extraction kit before SDS–PAGE. Proteins from different fractions were loaded onto an SDS–PAGE gel and subjected to electrophoresis. The proteins were subsequently transferred to a PVDF membrane. The membrane was blocked with 5% skim milk in TBST (pH 7.2) for 2 h at room temperature (RT). The membrane was then incubated with mouse anti-rMS087 polyclonal antiserum (1:10,000) in 5% skim milk diluted with TBST overnight at 4°C to block nonspecific binding. The samples were subsequently incubated with HRP-conjugated goat anti-mouse IgG (H + L) (1:10,000) (ABclonal, Wuhan, Hubei, China) at RT for 1 h. The protein bands were visualized using an ultra-enhanced chemiluminescence (ECL) reagent (Biosharp, Hefei, Anhui, China).

### 2.6 Optimization of the ELISA procedure and working conditions

Three *M. synoviae*-negative serum samples were collected from broilers, three *M. synoviae*-positive serum samples were obtained from broilers, and three *M. synoviae*-positive serum samples were drawn from laying hens. The status of the sera was confirmed with an IDEXX *M. synoviae* ELISA antibody test kit. Therefore, in this assay, three serum samples were used as negative controls and six serum samples were designated positive controls according to the results from the commercial ELISA antibody test kit.

Briefly, the wells of microtiter plates (Corning Incorporated, Kennebunk, ME, USA) were coated with 100 μL of rMS087 protein at concentrations from 0.05 μg/mL to 8 μg/mL in 0.05 mol/L carbonate buffer (pH 9.6) at 37°C for 1 h and then at 4°C overnight. After the unbound antigen was discarded, the wells were washed five times with PBS containing 0.05% Tween-20 (PBST). Nonspecific binding was blocked by incubation with 200 μL of PBST, 1% BSA, 1% ovalbumin (OVA), 2.5% skim milk, or 1% gelatin at 37°C for 0.5 h to 4 h. After five washes with PBST, 100 μL of each serum sample (diluted from 1:50 to 1:8,000 in blocking buffer) was added and incubated at 37°C for 0.5 h to 3 h. After five washes, 100 μL of HRP-conjugated goat anti-chicken IgG(H + L) secondary antibody (Bioss, Beijing, China) (diluted from 1:5,000 to 1:640,000) was added and incubated at 37°C for 0.5–3 h. After washing with PBST, 50 μL of substrate A (100 mL H_2_O containing 2.72 g of anhydrous sodium acetate, 0.35 g of citric acid monohydrate, 0.06 mL of 30% hydrogen peroxide) and substrate B (100 mL H_2_O containing 0.04 g of EDTA·Na_2_, 0.2078 g of citric acid monohydrate, 10 mL of glycerol, 0.0391 g of TMB·2HCl) (39) were added, the mixture was incubated at RT for 5–30 min, and the reaction was ended with the addition of 50 μL of 2 mol/L H_2_SO_4_. The optical density at 450 nm (OD_450_) was recorded with an ELISA plate reader (Thermo Fisher Scientific, Ratastie 2, FI-01620 Vantaa, Finland). Each experiment was performed at least three times, and all samples were assayed in triplicate. The working conditions were optimized by determining the highest *M. synoviae*-positive (P)-to-negative (N) serum OD_450_ ratios.

### 2.7 Calculation of the cut-off value

Three hundred and sixty-eight chicken serum samples were tested via the ELISA method established in this study, with three replicates for each serum sample. A receiver operating characteristic (ROC) curve was generated via GraphPad Prism 8.0 software on the basis of the average OD_450_ values determined for the serum samples, with the value generated at the maximum value of the Youden index was set as the cut-off value (40, 41).

### 2.8 Evaluation of reproducibility

Reproducibility, i.e., intra- and inter-assay variation, between runs was determined as described previously (39), with minor modifications. In brief, four *M. synoviae*-negative and four *M. synoviae*-positive serum samples identified by both the commercial ELISA antibody test kit and the rMS087-based ELISA antibody test method were selected randomly for reproducibility experiments. Five replicates of each sample in the same batch were chosen for the intra-assay (within plate) variation assessment, and three plates from different batches were chosen for the inter-assay (between runs) variation assessment. The coefficient of variations (CVs) were calculated.

### 2.9 Cross-reactivity with positive sera against other avian pathogens

The cross-reactivity of the established ELISA method with other avian pathogen-positive sera was investigated by using positive sera against *M. gallisepticum*, *Salmonella* Pullorum-Gallinarum, *A. paragallinarum* serovars A, B, and C, Newcastle disease virus, and avian influenza virus subtypes H5, H7 and H9. Two *M. synoviae*-negative and two *M. synoviae*-positive serum samples confirmed by both the IDEXX *M. synoviae* ELISA antibody test kit and the ELISA antibody test method established in this study were used as controls.

### 2.10 Maximum dilution of sera

Five *M. synoviae*-positive serum samples were diluted with blocking buffer as follows: 1:500, 1:1,000, 1:2,000, 1:4,000, 1:8,000, 1:16,000, 1:32,000, and 1:64,000. Then, ELISA was carried out with the optimal working conditions except for the optimal dilution of the serum. The maximum dilution of sera for the ELISA was assessed according to the cut-off value.

### 2.11 Serum test

Anti-*M. synoviae* antibodies were tested in 368 chicken serum samples via both a commercial ELISA antibody test kit and the rMS087-based ELISA antibody test method, with three replicates for each serum sample.

## 3 Results

### 3.1 Bioinformatic analysis

The full length of the *ms087* gene in the *M. synoviae* CQTL01 strain was 606 bp, and the gene did not contain a TGA codon, encoding tryptophan in *Mycoplasma*. The amino acid sequence shared over 97% homology with proteins of *M. synoviae* strains deposited in the GenBank database and had no more than 40% homology with the proteins of other *Mycoplasma* species according to the BLAST analysis (Supplementary Figure S1). For example, the amino acid sequence of *M. synoviae* had no more than 32% homology with the amino acid sequence of *M. meleagridis* and had no more than 21% homology with the amino acid sequence of *M. canis*. The ORF was predicted to encode a protein containing 201 amino acids with a MW of 23 kDa. The amino acid sequence of the MS087 protein was predicted to possess a signal peptide cleaved by signal peptidase I of the Sec translocator (the cleavage site is between amino acid positions 24 and 25) but lacked a transmembrane domain. Both CELLO v.2.5 and Gpos-mPLoc calculated that MS087 could be secreted by the organism as an extracellular protein. However, MS087 was predicted to have an antigenicity score of 0.3061 (the threshold score for antigenicity is 0.4).

### 3.2 Expression and purification of the rMS087

**Figure 1:**
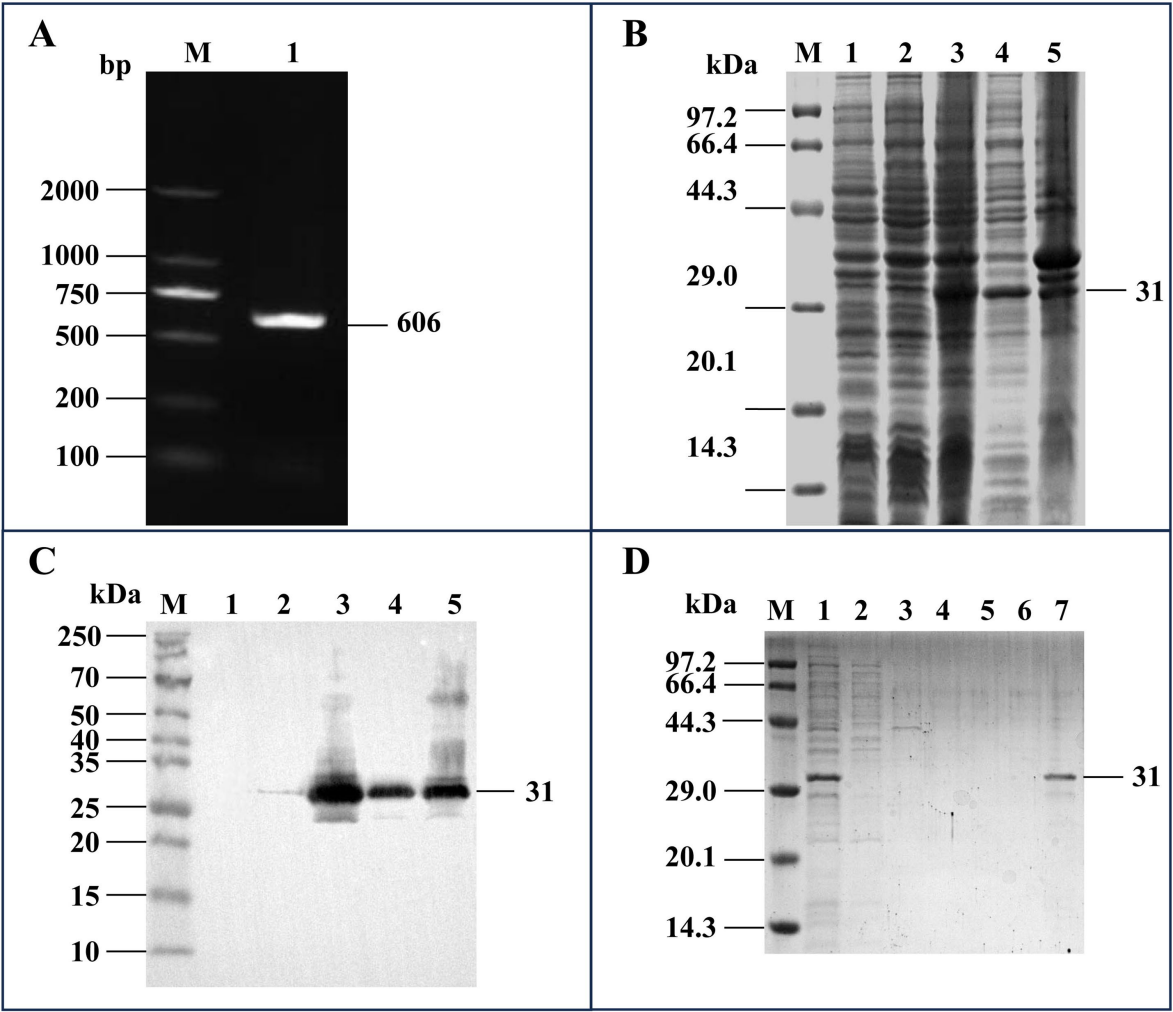
Cloning of the *ms087* gene and expression and purification of the rMS087 protein. (A) PCR amplification of the *ms087* gene by using the genomic DNA of the *M. synoviae* CQTL01 strain as the template. The electrophoresis was performed via an agarose gel. Lane M: DNA marker; lane 1: *ms087* gene. (B) Identification of the expression form of rMS087 in recombinant bacteria by SDS–PAGE. (C) Identification of the expression form of rMS087 in recombinant bacteria by Western blotting with a mouse anti-His tag antibody. (B,C) Lane M: protein marker; lane 1: *E. coli* BL21 (DE3) containing the pET-30a(+) plasmid; lane 2: total cell lysate of the recombinant bacteria without IPTG induction; lane 3: total cell lysate of the recombinant bacteria induced by IPTG; lane 4: the supernatant of the cell lysate of the recombinant bacteria induced by IPTG; lane 5: the precipitate of the cell lysate of the recombinant bacteria induced by IPTG. (D) Recombinant MS087 protein purified by Ni^2+^ affinity chromatography. The supernatant of the cell lysate was loaded onto (Lane 1) and flowed through (Lane 2) the column. The column was subsequently washed with a linear imidazole gradient of 0.1 mol/L (Lane 3), 0.2 mol/L (Lane 4) and 0.3 mol/L (Lane 5), and the purified protein was collected (Lane 6). (B–D) Five microliters of sample (1 mg/mL) was loaded into each lane of the SDS–PAGE gel, and the proteins in the SDS–PAGE gel were stained with Coomassie Brilliant Blue staining (B,D).

The full-length *ms087* gene was amplified from the CQTL01 strain via PCR (Figure 1A) and then cloned and inserted into the pET-30a(+) vector, which was subsequently successfully transformed into *E. coli* BL21(DE3) competent cells. After IPTG induction of the transformed cells, His-tagged rMS087 was expressed in soluble and inclusion body forms by the recombinant bacteria harboring the *ms087* gene (Figures 1B,C) and the purity of soluble rMS087 after purification was confirmed by SDS–PAGE (Figure 1D).

### 3.3 Production of polyclonal antiserum against rMS087

**Figure 2:**
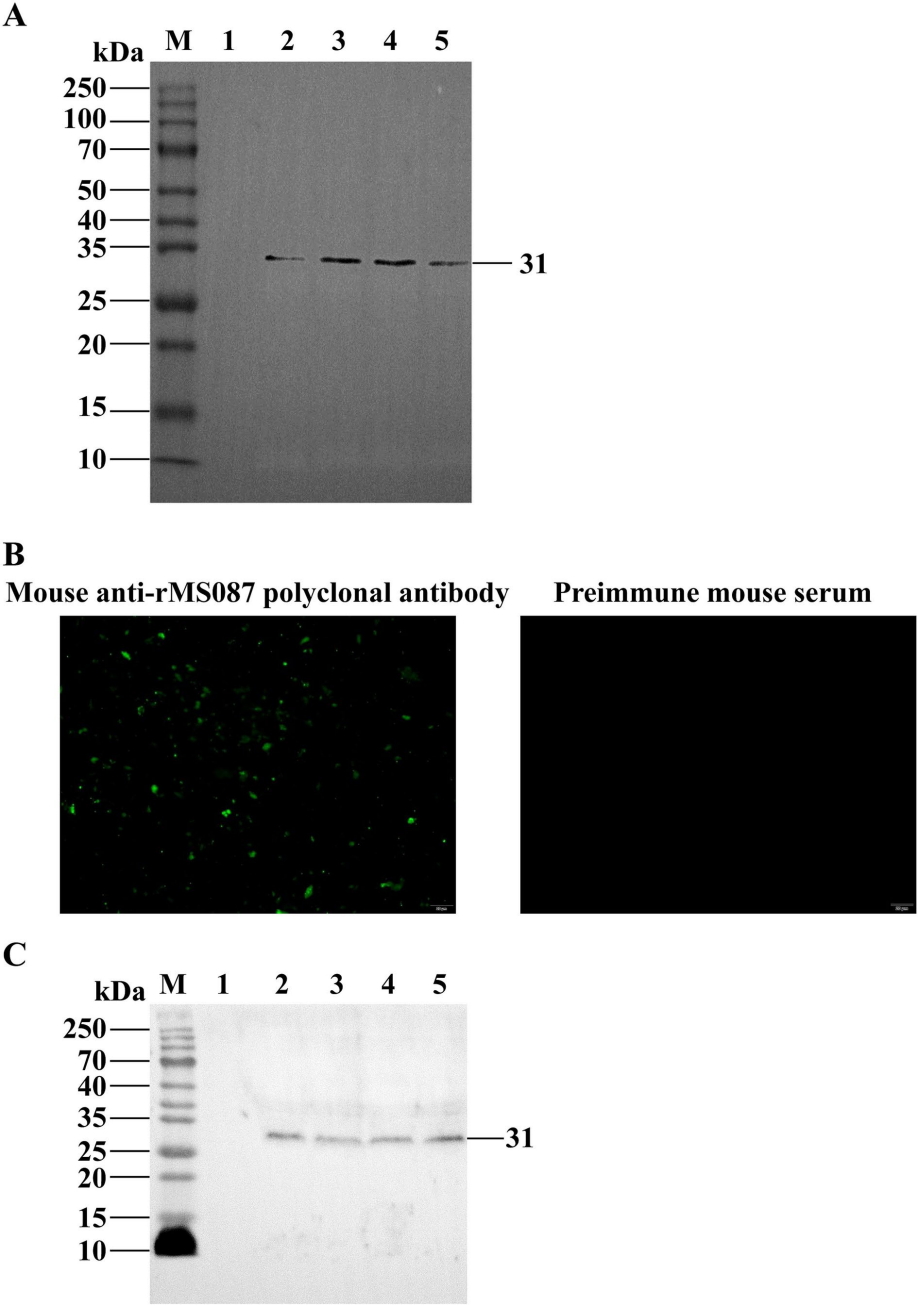
Identification of the subcellular localization of MS087 in *M. synoviae* by IFA and Western blotting. (A) Identification of the expression form of rMS087 in recombinant bacteria by Western blotting with a mouse anti-rMS087 antibody. Lane M: protein marker; lane 1: *E. coli* BL21 (DE3) containing the pET-30a(+) plasmid; lane 2: total cell lysate of the recombinant bacteria without IPTG induction; lane 3: total cell lysate of the recombinant bacteria induced by IPTG; lane 4: the supernatant of the cell lysate of the recombinant bacteria induced by IPTG; lane 5: the precipitate of the cell lysate of the recombinant bacteria induced by IPTG. (B) MS087 was localized on the surface of the *M. synoviae* strain CQTL01. (C) Analysis of the localization of MS087 by Western blotting. Lane M: protein marker; lane 1: whole cells; lane 2: cytoplasmic fraction; lane 3: membrane fraction; lane 4: culture medium supernatant. (A,C) Five microliters of sample (1 mg/mL) was loaded into each lane of the SDS–PAGE gel.

Purified rMS087 was used to generate rMS087 antiserum by immunization of BALB/c mice. The titers of the anti-rMS087 antibody in the sera of 5 mice were 1:1,024,000, 1:512,000, 1:256,000, 1:25,600, and 1:512,000. Antiserum with an antibody titer of 1:1,024,000 was used randomly for the subsequent experiments, and this antiserum could react with rMS087 of the recombinant bacteria (Figure 2A).

### 3.4 Distribution of MS087 in *M. synoviae*

IFA and Western blotting were performed to determine the distribution of MS087 in *M. synoviae* via the mouse anti-rMS087 polyclonal antiserum. In the suspension IFA, green fluorescent puncta were observed on the surface of the CQTL01 strain after incubation with mouse anti-rMS087 serum, whereas no fluorescent signal was observed after incubation with preimmune mouse serum (Figure 2B). These results implied that MS087 was localized on the surface of *M. synoviae*. The distribution of MS087 in *M. synoviae* was subsequently analyzed via Western blotting (Figure 2C). The mouse anti-rMS087 serum reacted with whole cell proteins, as well as membrane, cytoplasmic and extracellular proteins, at approximately 31 kDa, suggesting that MS087 is present in both the membrane and cytoplasm of *M. synoviae* and can even be secreted from the organism.

### 3.5 Optimization of the working conditions of the rMS087-based ELISA method for the detection of antiserum against *M. synoviae*

**Figure 3:**
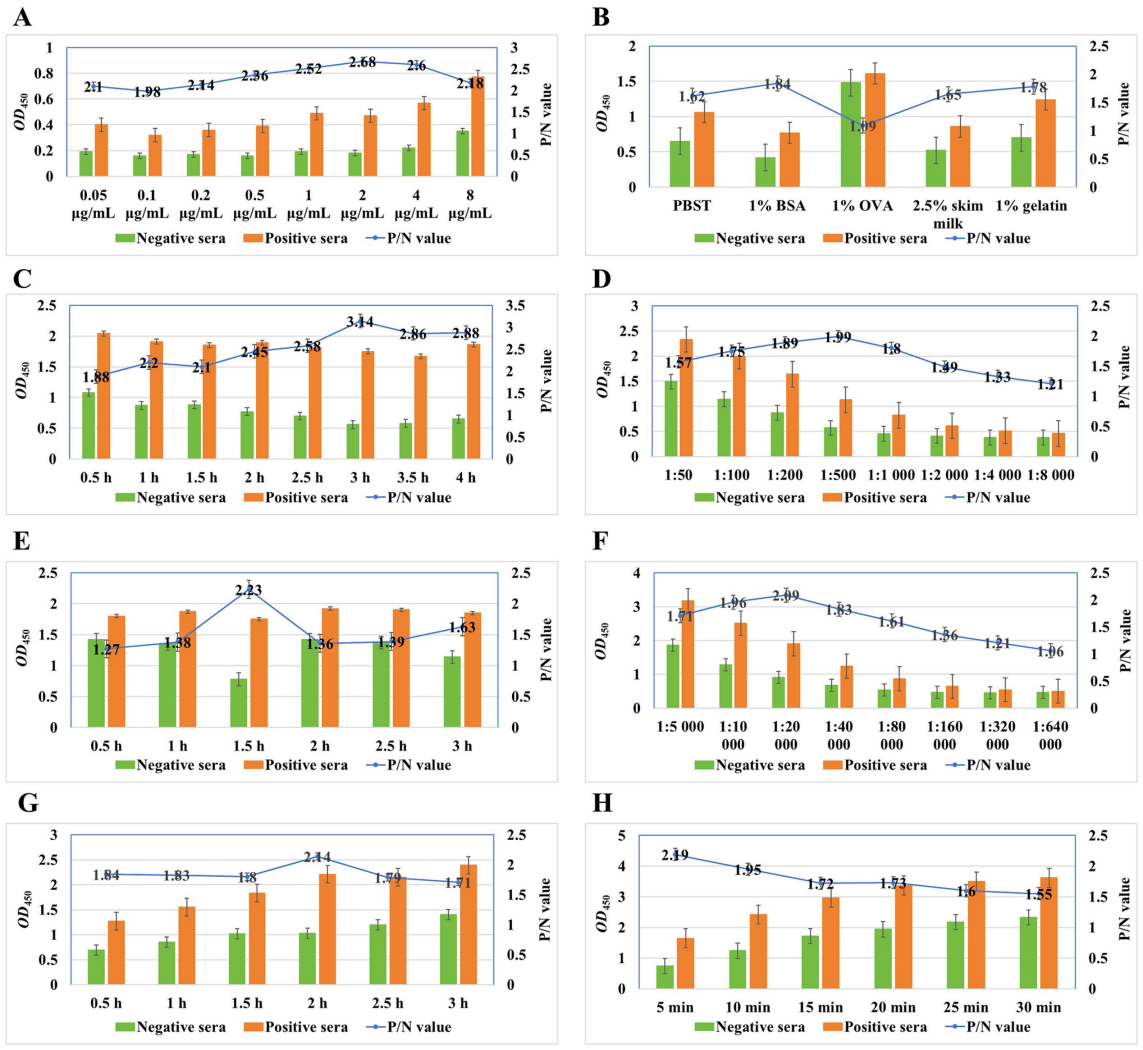
Optimal working conditions of the ELISA method for detection of anti-*M. synoviae* antibodies on the basis of the rMS087 protein. The optimal antigen concentration was 2 μg/mL in the coating buffer (A). The optimal blocking buffer was 1% BSA dissolved in PBS (B), and the optimal incubation duration for the blocking step was 3 h (C). The optimal dilutions of serum and secondary antibodies were 1:500 (D) and 1:20,000 (F) diluted in blocking buffer. The optimal incubation duration for the serum and secondary antibodies were 1.5 h (E) and 2 h (G), respectively. The optimal colorimetric reaction duration was determined after exposure to the substrate solution for 5 min (H).

Optimization of the working conditions for the detection of anti-*M. synoviae* antibodies in chicken sera involves the optimal antigen concentration, blocking buffer, blocking duration, serum dilution, serum incubation duration, secondary antibody dilution, secondary antibody incubation duration, and colorimetric reaction duration. As shown in Figure 3A, 100 μL of purified rMS087, adjusted to a concentration of 2 μg/mL with 0.05 mol/L carbonate buffer, was added to each well. After the unbound antigen was discarded, 200 μL of 1% BSA diluted with PBS (Figure 3B) was used to block nonspecific binding at 37°C for 3 h (Figure 3C). The optimal dilution of serum with 1% BSA was 1:500 (Figure 3D), and the optimal incubation time for the serum samples was 1.5 h (Figure 3E). As shown in Figure 3F, the P/N ratio increased with increasing HRP-conjugated goat anti-chicken IgG (H + L) secondary antibody dilution, reached a maximum at 1:20,000 and subsequently decreased with increasing serum dilution. Moreover, the highest P/N ratio was obtained after incubation with the secondary antibody for 2 h (Figure 3G). Finally, the highest P/N value was obtained when the enzyme reacted with the substrate for 5 min (Figure 3H).

### 3.6 Cut-off values for scoring ELISA-tested chicken sera

One hundred and fifty-eight serum samples from broilers and 210 serum samples from layers were assessed via the *M. synoviae* ELISA antibody method based on rMS087. The ROC curve was generated on the basis of the values obtained from 368 serum samples, with the maximum value of the Youden index as the cut-off value. The maximum Youden index was 0.958, and the cut-off value was 0.5, indicating that when the OD_450_ value of the serum to be tested was ≥0.50, the sample was considered as positive for anti-*M. synoviae* antibodies; when the OD_450_ value was <0.50, the sample was considered as negative for anti-*M. synoviae* antibodies.

### 3.7 Reproducibility, cross reactivity and maximum serum dilution of the *M. synoviae* ELISA antibody test method

The reproducibility of the rMS087-based ELISA was assessed by determining the intra- and inter-assay variation. The intra-assay CVs of 4 negative serum samples and four positive serum samples ranged from 1.32 to 5.50%, and the inter-assay CVs of these samples ranged from 4.01 to 8.77%, suggesting that the ELISA results are reproducible, yielding low and acceptable variation.

**Figure 4:**
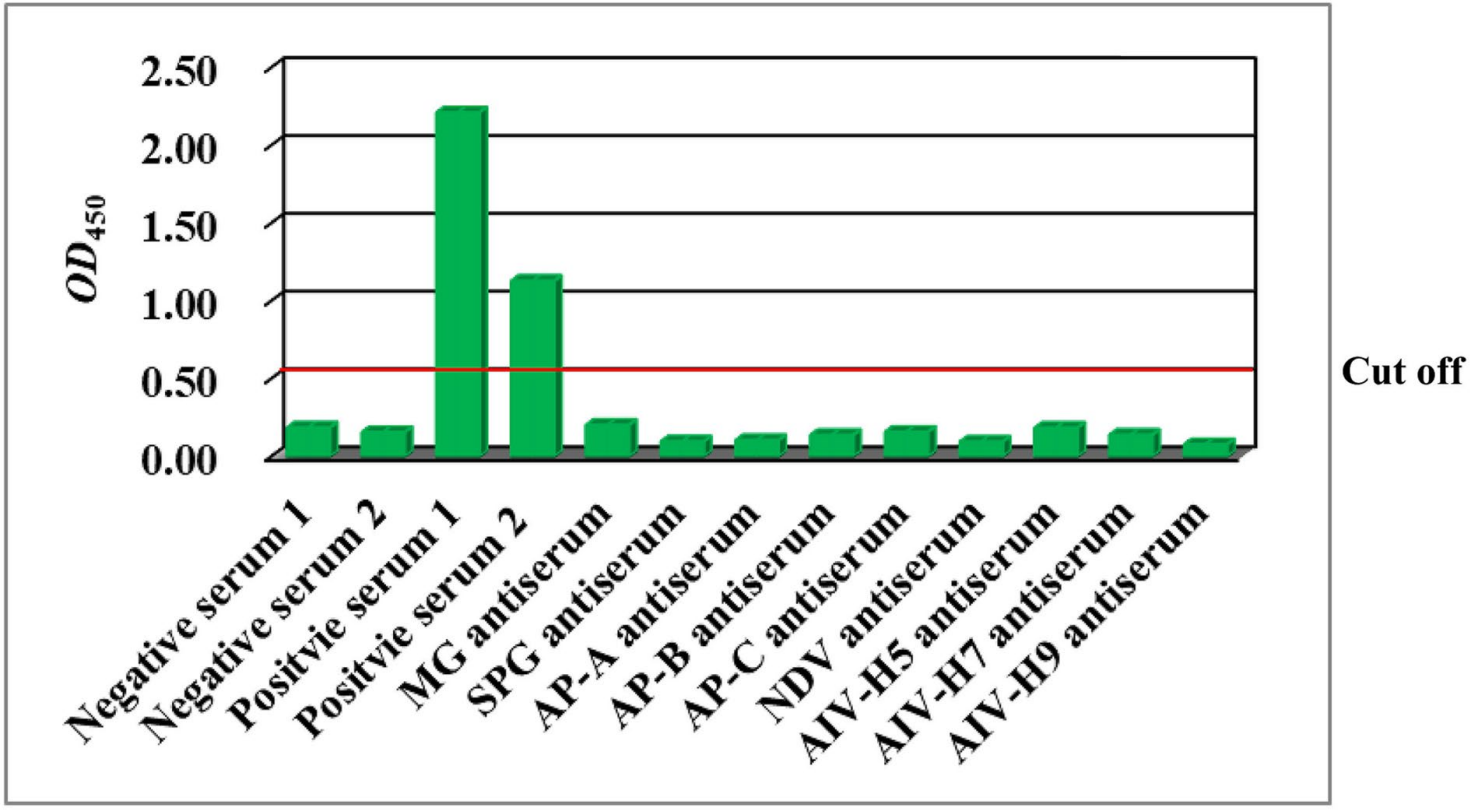
Results of the detection of positive sera against avian pathogens via the rMS087-based ELISA antibody test method. MG: *M. gallisepticum*; SPG: *Salmonella* Pullorum-Gallinarum; AP-A: *A. paragallinarum* serovar A; AP-B: *A. paragallinarum* serovar B; AP-C: *A. paragallinarum* serovar C; NDV: Newcastle disease virus; AIV-H5: avian influenza virus subtype H5; AIV-H7: avian influenza virus subtype H7; AIV-H9: avian influenza virus subtype H9.

The ELISA exhibited no cross-reactivity with sera containing antibodies against nine avian pathogens (Figure 4). These data indicated that the ELISA was specific to anti-*M. synoviae* antibodies induced by natural infection and that there was no cross-reaction with antisera against other avian pathogens.

**Figure 5:**
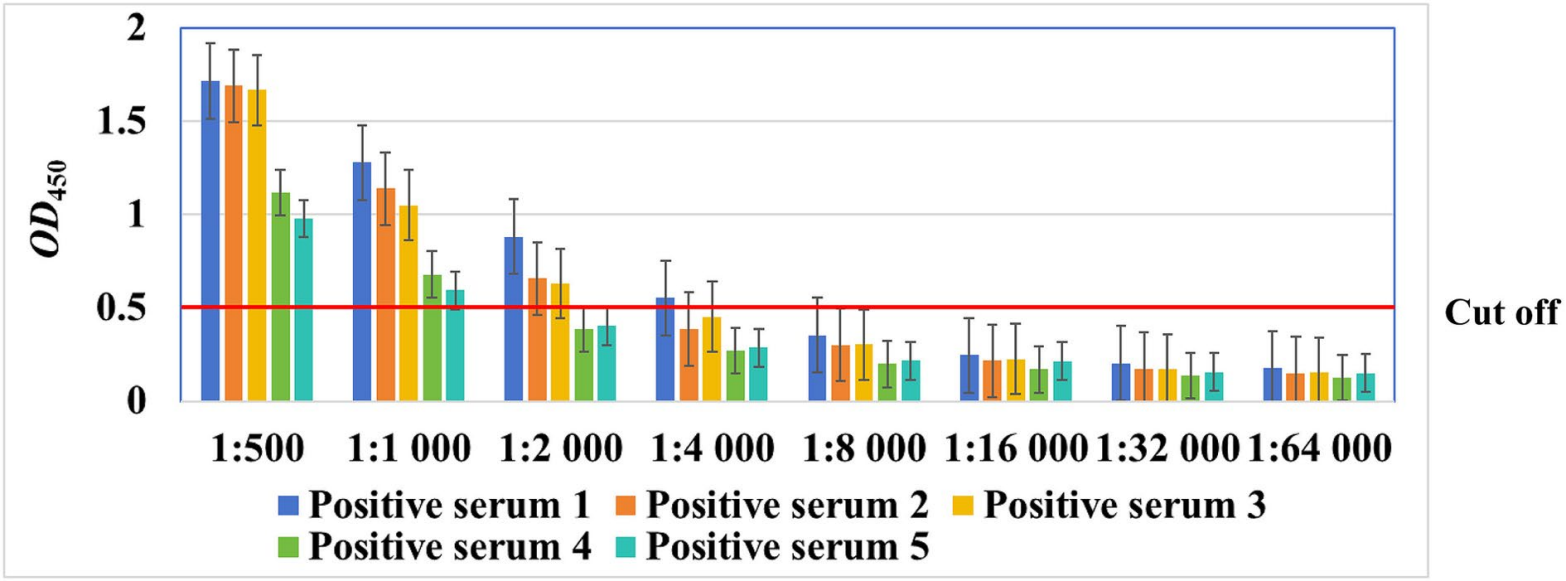
The maximum dilution ratio of chicken sera for detection by the rMS087-based ELISA antibody test method.

As shown in Figure 5, with increasing dilution ratio of the serum, the OD_450_ value decreased gradually. Five serum samples at a dilution of 1:1,000, three at a dilution of 1:2,000, and one at a dilution of 1:4,000 were considered positive, whereas no serum sample at a dilution of 1:8,000 or more were considered positive, suggesting that the serum can be diluted up to 1,000 times when using this method.

### 3.8 Comparisons with different detection methods

Serum samples collected from two slaughterhouses and five poultry farms were tested via a commercial ELISA kit and the rMS087-based ELISA method. The results are shown in Table 2. The results of the commercial ELISA kit and the rMS087-based ELISA were inconsistent in 28 samples. The total agreement between the commercial ELISA kit and the rMS087-based ELISA was 92.4%, with the sensitivity and specificity of the rMS087-based ELISA being 93.2% and 91.5% compared to those of the commercial ELISA kit, respectively. However, the rMS087-based ELISA method (52.4%, 193/368) could detect more M. synoviae-positive serum samples than commercial ELISA kit (51.9%, 191/368).

**Table 2:** Detection of 368 chicken serum samples via a *M. synoviae* commercial ELISA antibody test kit and the rMS087-based ELISA antibody test method.

Commercial ELISA antibody test kit	ELISA antibody test method based on rMS087		Total
	Positive	Negative	
Positive	178	13	191
Negative	15	162	177
Total	193	175	368

## 4 Discussion

Accurate detection is a prerequisite for the effective prevention and control of *M. synoviae* infection. In modern poultry production, molecular detection and serological investigations are the most commonly used methods for infectious disease diagnosis. Owing to the sampling sites (upper respiratory tract) used in live poultry (42, 43), the sensitivity of molecular methods for detecting *M. synoviae* is limited. Serological methods, especially ELISA, make the detection of *M. synoviae* infection more convenient and inexpensive and have a higher detection rate than real-time PCR does (42), even if antibodies cannot be detected in the early stage of infection (31).

The ELISA methods established previously have shown cross-reactivity with antisera against other avian *Mycoplasmas* due to the use of whole bacterial cells or membrane proteins (21, 24–26) or have shown reduced sensitivity because of variable amino acid sequences in selected proteins (27, 28).

MS087, which contains 201 amino acids, was predicted to be 23 kDa even if its signal peptide was not cleaved. However, the MW of MS087 in *M. synoviae* (Figure 2C) or recombinant bacteria (Figure 1) is 31 kDa. The actual MW of MS087 is larger than the predicted MW. One possible reason might be due to inaccurate prediction of online bioinformatics. The second possible reason was that the protein undergone posttranslational modification, such as acetylation (44), lysine methylation (45), etc.

Through BLAST analysis, we showed that MS087 was predicted to be an F1-like ATPase-associated subunit localized on the cell membrane (32), widely expressed in *M. synoviae* and had a highly conserved amino acid sequence. Our study showed that the protein was localized in both the cytoplasm and the membrane and was even secreted from the organism in our study. The data mentioned above indicate that the MS087 protein has the potential to serve as a good coating antigen for the development of an ELISA method to detect *M. synoviae* infection in chicken flocks.

We visited several layer farms. The breeders reported that laying hens that produced eggs with EAA did not develop arthritis in either layer pullet flocks or laying hen flocks. These findings suggest that different strains of *M. synoviae*, which cause arthritis in broilers and the production of eggs with EAA in laying hens, may have different tissue tropisms, although this speculation has not been proven. Therefore, in the process of establishing this method, we used positive control sera against *M. synoviae* derived from broilers and laying hens.

A cross-reactivity test revealed that the ELISA method based on rMS087 did not react with antisera against other major avian pathogens. Both the specificity (91.5%) and the sensitivity (93.2%) of the rMS087-based ELISA were greater than 90%. Whereas, the specificity and the sensitivity of the LP78-based ELISA were 94.1% and 85.7%, respectively (31). Moreover, the ELISA method based on rMS087 (52.4%, 193/368) detected more positive sera against M. synoviae compared to the commercial kit (51.9%, 191/368), and the rLP78-based ELISA (61.8%, 105/170) detected fewer positive sera against M. synoviae than the commercial kit (70.0%, 119/170) (31). This difference may be because the LP78 protein is a membrane protein, while the MS087 protein not only exists on the membrane but can also be secreted out of cells, continuously stimulating the Th2 cell response and inducing the production of more antibodies against MS087.

There are four main types of *Mycoplasmas* that cause diseases in poultry, namely *M. gallisepticum*, *M. synoviae*, *M. meleagridis*, and *M. iowae*. *M. meleagridis* and *M. iowae* are the turkey pathogens. The purpose of this study was to establish an ELISA for the detection of anti-*M. synoviae* antibodies in chicken sera. Therefore, when determining whether there is cross-reactivity in the established detection method, only *M. gallisepticum* antibodies were used.

In subsequent work, we will use different *M. synoviae* strains to infect specific pathogen-free (SPF) white Leghorn-type chickens and *M. synoviae*-free layers to determine when the rMS087-based ELISA can detect anti-*M. synoviae* antibodies after infection. These findings can provide data to support the early diagnosis of *M. synoviae* infection. On this basis, the sensitivities of the rMS087-based ELISA and real-time fluorescence quantitative PCR will be compared.

## 5 Conclusion

In summary, this study established an ELISA method based on the secretory protein MS087 of *M. synoviae*, which has good specificity and sensitivity, and MS087could be used as a coating antigen for the detection of serum antibodies against *M. synoviae* in chicken flocks.

## Data Availability

The datasets presented in this study can be found in online repositories. The names of the repository/repositories and accession number(s) can be found at: https://ngdc.cncb.ac.cn/gsa/search?searchTerm=CRR1309196.

## Footnotes

^1^ https://www.detaibio.com/sms2/protein_mw.html

^2^ https://blast.ncbi.nlm.nih.gov/
