# Supplementary figures and images for: Recombinant MS087-based indirect ELISA for the diagnosis of Mycoplasma synoviae
Source: Front Vet Sci. 2024 Oct 29;11:1472979. doi: 10.3389/fvets.2024.1472979 (PMC11555648; doi:10.3389/fvets.2024.1472979)

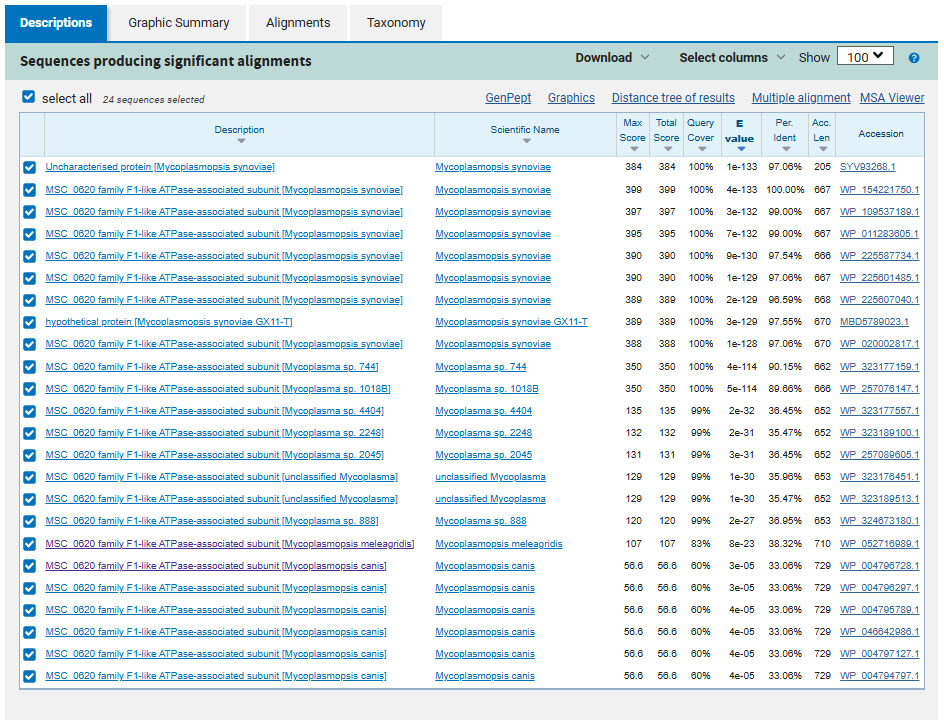

Supplement: Supplementary file 1 [file Image_1.jpeg]
